# Mapping the Transition of Adolescents to Adult HIV Care: A Mixed-Methods Perspective from the Cape Town Metropole, South Africa

**DOI:** 10.3390/tropicalmed10010005

**Published:** 2024-12-24

**Authors:** Charné Petinger, Brian van Wyk, Talitha Crowley

**Affiliations:** 1 School of Public Health, University of the Western Cape, Cape Town 7535, South Africa; bvanwyk@uwc.ac.za; 2 School of Nursing, University of the Western Cape, Cape Town 7535, South Africa; tcrowley@uwc.ac.za

**Keywords:** ALHIV, HIV, transitions of care, transfer pathways, mixed methods

## Abstract

(1) Background: Globally, an estimated 1.7 million adolescents (aged 10–19 years) were living with HIV in 2023, with 82% residing in sub-Saharan Africa. For ALHIV, transitioning to adult care involves assuming responsibility for their own health and disease management, posing significant challenges to persistent engagement in care. There is a paucity in health policies guiding this transition in many sub-Saharan African countries. Overburdened and poorly functioning health systems struggle to provide optimal care for ALHIV amidst the rising HIV pandemic in this priority population. (2) Methods: This study employed a mixed-methods design, comprising a descriptive qualitative study with healthcare workers and managers and a cross-sectional survey to examine the practices and pathways in which the transition to adult HIV care occurs in the Cape Town Metropole, South Africa. (3) Results: We delineate three distinct ways in which transition occurs (transfer-only, adolescent-friendly, and supportive transition). A successful transition involves a sufficient level of self-management of their chronic condition and healthcare journey, which is preceded by adequate preparation pre-transition, and the monitoring of engagement post-transition. This ideally requires developing relevant health policies and implementing guidelines signaling political will and providing the impetus and agency of implementation at the service level in South Africa.

## 1. Introduction

Globally, it is estimated that 1.7 million adolescents (aged 10–19 years) are living with HIV in 2023, with 82% residing in Sub-Saharan Africa [1,2]. The successful roll-out of antiretroviral therapy (ART) and the development of more efficacious antiretroviral regimens (ARVs) contributed to positive health outcomes for children who were vertically infected with HIV, which, along with the growing number of new HIV infections, resulted in a rising number of adolescents living with HIV (ALHIV) [1,3]. Adolescence is a period characterized by a myriad of physical, social, and emotional changes and development [4], which makes it particularly difficult for ALHIV living with a chronic condition to successfully navigate it [4]. Further, ALHIV are challenged by wide-spread social and self-stigma in the school, work, and community environments, which hinders the attendance of clinic appointments, and complicates the persistent adherence to medication [4,5,6]. In addition, older ALHIV are expected to increasingly take personal responsibility for their chronic condition, which invariably leads to lower rates of adherence and interruptions in care, to the detriment of their overall health and mental well-being [5,7,8].

It is estimated that approximately 320,000 ALHIV will transition from pediatric to adult antiretroviral therapy (ART) care by 2028 in South Africa [9,10]. In South Africa, ALHIV are expected to transfer from pediatric to adult HIV care around the ages of 10–13 years [11,12], to be advantaged by more simplified treatment regimens and greater options for treatment [13,14]. The transition process can be described as a shift from caregiver-led treatment (in pediatrics) to autonomous treatment-taking and care (in adult care) [15]. Additionally, the healthcare transition is expected to involve a negotiation between the patient, their parents or caregivers, the pediatric healthcare provider, and the adult healthcare provider to ensure that autonomous health-related decision-making is achieved [16]. It is important to note the distinction between transfer and transition, where transfer refers to the actual point when the adolescent is moved to a different healthcare provider [17]. The change in care and providers differs, as, in pediatric settings, ALHIV are provided with individualized care and closer relationships with their providers, whereas adult care can be conceptualized as independent care [15,18]. Thus, ALHIV are expected to move from a familiar and supportive system towards one that may be unfamiliar, where they must be independent, and in the context of an overburdened health service—particularly, in the South African context with a high HIV prevalence [19,20].

For ALHIV, transitioning to adult care involves taking charge of their own health and disease management, which raises the personal challenge of persistent engagement in care [3]. When the transition is executed poorly, it results in interruptions in care and adverse health outcomes for the adolescent such as virological failure and a higher risk of mortality [19,21]. The above-mentioned risks occur when ALHIV are transitioned before they are ready or not adequately prepared or guided in the process of assuming self-responsibility for their care [12,21]. A recent review reports that there are few transition policies and guidelines in place at both the national and operational levels [22]. Overburdened and poorly functioning health systems and models of care for ALHIV are extant in the African context, where the brunt of the HIV pandemic amongst adolescents is situated. This can, therefore, have a direct influence on their engagement (or lack thereof) in care, and, ultimately, overall health outcomes [3,23].

Comprehensive and structured transition policies and guidelines that are adolescent-sensitive positively impact the health outcomes of ALHIV [24]. Services that are geared towards the needs of adolescents, such as supportive staff, flexible clinic hours outside of school, and services that offer peer networking are beneficial toward successful HIV care for ALHIV [7]. Supportive transition processes may increase adolescents’ knowledge and skills to manage their condition and increase the likelihood of adherence, retention in care, and viral load suppression [12,24]. In South Africa, there are no national guidelines or protocols to manage the transition in different healthcare contexts [25]. The National Strategic Plan (NSP) [26] only acknowledges the transition process as a whole, whereas the HIV clinical guidelines only prioritize the change in regimens [27,28]. Guidelines for transitioning ALHIV have been developed for high-resource settings such as the USA [19] but are relatively sparse in low-resource settings such as Africa. Two notable exceptions are transition protocols and support interventions that were implemented in Kenya [29] and South Africa [30]. In Kenya, the ATTACH trial was developed to provide healthcare workers and adolescents with disclosure and transition tools [29]. The InTSHA intervention was implemented in South Africa as trained peer facilitated group discussions to foster shared experiences and networking. The Elizabeth Glazer Pediatric AIDS Foundation developed a toolkit to be used by healthcare providers for transitioning ALHIV, but its implementation in high-HIV-prevalent, low-resource settings is not clear [31]. Sam-Agudu et al. [32] developed a coordinated transition protocol in Nigeria to provide this additional transition support that can be integrated within an African healthcare system. Jegede and van Wyk [22] found that the integration of peer support and navigation into transition protocols will ensure better post-transition outcomes through facilitating the transition process.

The transition process should comprise support and care being provided to adolescents prior to, during, and after transitioning to adult care. This includes adequately informing ALHIV on what the transition entails, assessing their knowledge of HIV and self-management skills, bolstering communication between the pediatric and adult care providers, addressing fears relating to adult services, and providing additional support to ensure they remain engaged in care after they have been transferred to adult care [19,33,34,35].

ALHIV in low- and middle-income countries are at risk of developmental, psychosocial, and comorbidity issues, making them a particularly vulnerable group, and acknowledged as a priority population in HIV control [3]. In many low-resource contexts, the healthcare systems are not tailored to address the specific structural requirements and unique needs of ALHIV [3,36]. In South Africa, which is a middle-income country with huge discrepancies between low- and high-resource settings, a lack of structure and ineffective communication between pediatric and adult healthcare providers are a major barrier [36]. There is a need to garner a deeper understanding of existing transition practices and pathways, ensuring that these are tailored to the requirements of ALHIV, and aligned with the available resources within the contexts of low- and middle-income countries [25,37,38]. There is also a lack of written protocols to inform evidence-based and well-established transition guidelines [25,39]. The development of national-level policies tailored to the unique experiences and needs of ALHIV who are transitioning from pediatric to adult HIV care is necessary, as the current practices within South Africa are found to result in poor engagement in care and an inconsistent timing of the transition [10,25]. Moreover, there is a paucity in the evidence for the occurrence of the transition to adult HIV care specifically in the Western Cape, South Africa. Optimizing policies and practices would lead to successful health outcomes for ALHIV [10,40,41]. Thus, to improve the overall adherence to ART for ALHIV, care that is tailored towards their needs is necessary [42]. The aim of this study was to describe the pathways by which the transitioning of ALHIV from pediatric to adult HIV care takes place in the Cape Town Metropole, South Africa.

## 2. Materials and Methods

### 2.1. Overview

This study followed a mixed-methods study design with a predominantly qualitative descriptive study component, and a complementary cross-sectional survey to describe the services that are delivered to ALHIV in selected sites in the Cape Town Metropole. The direction of this study was informed by qualitative evidence synthesis examining evidence of transition experiences of ALHIV (unpublished) that was undertaken by the authors, which identified the structural facilitators and barriers in the transition process for ALHIV. A qualitative descriptive study provided a factual and straightforward description of experiences and perceptions on the practices of healthcare workers on transitioning ALHIV to adult HIV care [43,44]. This is because the purpose of descriptive studies is to describe the individual or event as it is in its nature. In this case, it can be used to accurately describe the practices of transition and the pathways in which it takes place in the public health system in the Cape Town Metropole [45]. Furthermore, it is flexible in a way where this research can obtain a rich amount of data, leading to comprehensive results to answer our inquiry into the practices and pathways wherein the transition to adult HIV care occurs [46]. The structured survey recorded the practices of services (pediatric, adolescent, and adult) provided to ALHIV across multiple facilities. Surveys are beneficial in mixed-methods research as it allows a richer and more comprehensive approach to the topic under investigation [47]. Survey research is useful as it can assess the topic of interest accurately, in this case, investigating the services being provided to ALHIV [48].

### 2.2. Study Setting

The Cape Town Metropole has a population of approximately 637,353 adolescents between the ages of 10–19 years as of 2020 [49]. Moreover, the Metropole is a highly burdened area of people living with HIV, with 540,000 people on ART in 2022 [50]. The study was conducted in public primary-, secondary-, and tertiary-level facilities in the Cape Town Metropole. Primary-level facilities included primary healthcare clinics and community health clinics [51]. Secondary-level facilities include regional hospitals providing specialist care, emergency services, in- and out-patient services, and pediatric and obstetric services [51]. Tertiary-level facilities include all levels of care, referral facilities, and specialized services [51].

### 2.3. Sampling and Participants

For the qualitative component, 16 participants were purposefully sampled, with the inclusion criteria of being healthcare workers or managers providing services to ALHIV within the public healthcare system in the Cape Town Metropole. After initial participants were identified, subsequent participants were recruited via snowball sampling from the initial participants. For the quantitative component, six participants were identified using maximum variability purposive sampling (one participant per facility). The sample size was pragmatically informed by the qualitative interviews with consideration of including the different levels of care and the provincial access granted to this study. The sample size was not powered to detect statistical significance, but to provide descriptive insights into the care provided at the facilities.

### 2.4. Data Collection

#### 2.4.1. Qualitative Data Collection

This study utilized individual, semi-structured interviews to collect qualitative data. The interviews were conducted by the researcher who was accompanied by her supervisors and another researcher for five of the interviews. Interviews lasted between 30–60 min and were conducted at a place and time most convenient for the participants. Data collection took place from February–August 2024. A semi-structured interview guide (see Supplementary File S1) was used to probe healthcare workers and managers on how transition takes place at their facilities, their understanding of successful transition, the challenges to deliver pediatric and/or adolescent HIV care, and how they sought to overcome these challenges in service delivery for ALHIV. The interviews were audio-recorded and transcribed verbatim by the researcher.

#### 2.4.2. Quantitative Data Collection

Quantitative data on services provided to adolescents living with HIV were collected through a structured survey questionnaire that was developed in a national survey in Nigeria [39]. The questionnaire comprised six sections: site profile, clinic services, adolescent care services, adolescent support group services, and transfer of adolescents from pediatric to adult clinic [39]. The survey tool was developed on RedCap software (Version 14.5.27), whereafter a link was sent to one participant per facility via email.

### 2.5. Data Analysis

#### 2.5.1. Qualitative Data Analysis

Transcriptions of the interviews were uploaded to and analyzed within the Atlas.Ti software (Version 24). The collected data were analyzed using reflexive thematic analysis, as developed by Braun and Clarke [52]. Reflexive thematic analysis was useful to comprehensively analyze data in a methodological manner, whilst maintaining the reflexive nature of the qualitative approach [52]. By following these steps and developing codes and themes, it allowed the researchers to answer the research questions accurately and descriptively [53].

#### 2.5.2. Quantitative Data Analysis

The survey data were extracted from RedCap into SPSS version 29 for analysis. Descriptive statistics were used—frequencies and data distributions—to describe whether there are adolescent-specific services, and if there are formal transition/transfer procedures and criteria within the facilities. Table 1 provides the site characteristics, and Table 2 describes characteristics of adolescent-specific services.

### 2.6. Rigor

Trustworthiness for the qualitative phase was maintained through the four concepts of Lincoln and Guba (1985): credibility, dependability, transferability, and confirmability [54,55,56]. Credibility is ensured through thick descriptions of the data, dependability is achieved through the consistency of the procedures utilized during data collection and the interview guide, rich descriptions of the participants’ experiences and contexts will inform transferability, and confirmability is ensured through evaluating researcher bias [43,55]. The researcher kept a reflective diary, which was shared with the co-authors, throughout the data collection process to ensure that trustworthiness was maintained. The reflective diary played a role in triangulation, whereby this assisted in the interpretation of the data from all authors, which, in turn, facilitated the process of identifying and developing the pathways discussed in the results of this study.

Validity and reliability for the quantitative phase was maintained through piloting the survey prior to mailing it to the participants to identify any potential issues and assess the readability of each question. Moreover, as this was a developed survey, validity was ensured by the authors to ensure that each question measures what it sets out to [39]. Reliability was maintained through sending the same link to all participants via email.

### 2.7. Ethics Considerations

This study forms part of a PhD project of the first author (CP), and ethics clearance was obtained at the registered university (BM23/6/5), and permission to access the facilities was granted by the Provincial Health Research Committee (WC_202308_043). An information sheet and consent form were provided to the participants for both the interviews and the survey prior to data collection. Confidentiality and anonymity were ensured throughout the process through the use of pseudonyms for the participants. The collected data were stored in a secured, password-protected file, with sole access to the researcher and her supervisors.

## 3. Results

### 3.1. Description of Participants

For the qualitative interviews, sixteen (16) participants were interviewed, all identifying as women. All participants are employed within the Cape Town Metropole, in public healthcare facilities, of which ten were in KESS, two in Eastern, three in Tygerberg, and one in the Mitchell’s Plain Sub-Structure. In terms of their designations, our participants comprised five medical officers, four pediatric medical officers, two HIV counsellors, one HAST (HIV, AIDS, STI, and TB) manager, two clinical nurse practitioners, and two who were employed as both clinical nurse practitioners and operational managers within their facility.

### 3.2. Description of Facilities

Six facilities were included in this study. As mentioned previously, all facilities fall in the Cape Town Metropole. There were four facilities in KESS, ranging from primary to secondary levels of care. We included one tertiary-level facility, situated in the Tygerberg Sub-Structure. Finally, a primary-level facility from the Mitchell’s Plain Sub-Structure was included.

### 3.3. Survey of Transition Mapping

Six key informants from six facilities completed the survey. Table 1 describes the characteristics of the included facilities and how many ALHIV are enrolled in care at these facilities. Table 2 illustrates the different services the facilities provide as well as what adolescent-friendly services they provide, how the transfer takes place, and if there are any criteria for transferring ALHIV to adult services. Only the facilities which have adolescent-specific transition and post-transition practices are included in the table below, as a stop action was placed on the survey if there are no dedicated adolescent and/or transition practices. As illustrated in Table 2, only three of the six facilities reported a routine practice for transitioning ALHIV to adult care, wherein one facility had a post-transition follow-up with the patients. The tertiary-level facility had five criteria to transfer adolescents, (age, readiness assessment, pregnancy, marriage, and viral load), whereas the secondary facility had one (age).

### 3.4. Mapping Transition

We identified three pathways—*transfer-only, adolescent-friendly*, and *supportive transition*—in which transition takes place within the public health facilities in the Cape Town Metropole. This was achieved through the thematic analysis of qualitative data, which was strengthened after the quantitative data were collected and analyzed.

**Figure 1:**
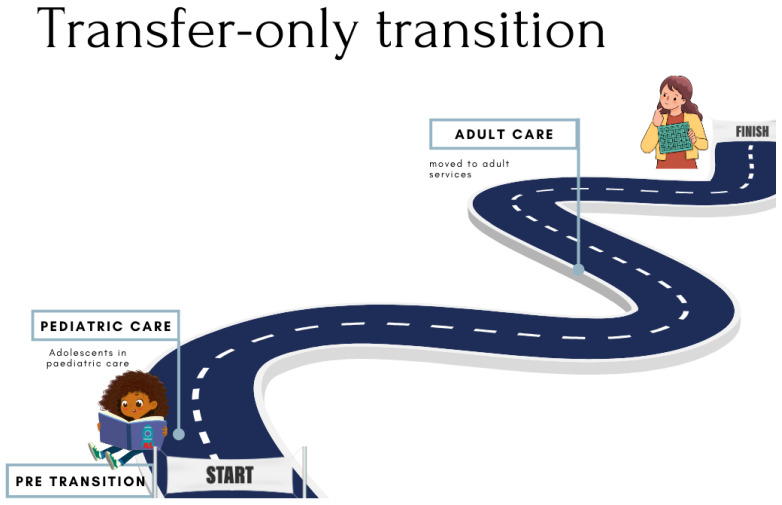
Transfer-only pathway.

Figure 1 illustrates the first pathway, that we termed *transfer only*. In this pathway (Figure 1), ALHIV on ART are moved from pediatric to adult HIV care with little to no preparation or assessment of readiness. Moreover, patients are either transferred outside of the facility to a primary healthcare clinic, or general adult HIV services within the same facility, but to a different provider. There also exists a lack of a dedicated adolescent-focused healthcare provider, space, and funding.

Whilst these facilities do not have adolescent-specific or -focused services such as youth clubs, the healthcare providers attempt to provide individualized care to children with HIV who grow up to become ALHIV.

*Unfortunately, when you come to a hospital and you come to an HIV clinic, then people can ask questions about your health and your HIV status. They aren’t necessarily going to ask you about everything else that’s happening in your life, exactly like, do you play hockey?*

(Participant J, pediatric medical officer)

Participant J explains that the care she, as an individual practitioner, can provide is more person-focused than what the adolescent may receive in adult services. Participant F confirmed that “*individualized attention just isn’t given in the adult services because of the patient load*” (Participant F, pediatric medical officer). As a result of this polarized choice between pediatric care and adult care, pediatric HIV doctors tend to hold on to adolescent patients and are reluctant to release them to adult care.

When asked about the option of providing adolescent-specific services, participants mentioned that the facility cannot offer these services:

*Forever it’s a struggle between who must take care of them* [adolescents] *when they have chronic diseases.*

(Participant K, pediatric medical officer)

This indicates an inattention towards adolescent services on a facility level, despite the providers’ ability to recognize the need for it. Participant F supports this:

*I mean, in America, there’s pediatrics and there’s adolescent health and there’s adult health. So there, there isn’t what we do have.*

(Participant F)

Thus, the participants show an awareness of the need for and advantages of adolescent-focused services in other contexts and conceded that there may be a deficit in their care offering to ALHIV in their facility. However, to be able to provide this, the facility requires a point person. Further to the facility-level challenges, the participants enunciate the lack of an “adolescent-trained” care provider that may address the apparent gap in the services provided to ALHIV.

*We need a person to come. We need someone that likes adolescents. We can’t force someone who doesn’t like adolescents.*

(Participant J)

The *transfer-only* transition not only explicates the nonexistence of adolescent-specific services being provided to ALHIV, but also a minimized transition process wherein there is no comprehensive discussion around what this transition in its entirety entails with the adolescent, as well as between the pediatric and adult HIV service providers. Thus, the transfer is carried out solely on the discretion of the pediatric medical officer.

Moreover, this may not always be a formal transfer, particularly in instances where the pediatric and adult doctors are in one facility. Participant E discusses that in this informal transfer:

*I kind of pick them up as problems and then I take them over. So, it’s often happened that I, we, they’ve kind of become mine by default with mine, I mean the adult clinic.*

(Participant E, medical officer)

Participant D furthers how this transfer is individualized and there is no one way of transitioning adolescents within this pathway:

*Then they* [pediatric doctors] *normally write in the file, transfer to adult clinic, and we then take over those kids, young adults. Sometimes they do it when they’re 17, sometimes they hang on to them till they’re 20.*

(Participant D, medical officer)

Whilst the facility and majority of staff remain the same for the adolescent patient, there is no acknowledgement of the specific care that should be given to adolescents and the current developmental period they are in.

It is evidenced that, even post-transfer, ALHIV tend to disengage from care and have a higher viral load, particularly in the South African context [6,12,57]. This requires a post-transfer check-in with the adolescent patient, to ensure that they remain in care. Participants mention their own regard for their previous patients:

*I’m always wondering, do they go? Is there any way that you can make sure they continue their service?*

(Participant K)

*So, it’s very difficult, like some kids do well when you transfer them out, and some don’t do well because there isn’t someone checking up on them all the time.*

(Participant J)

As a result of the lack of measures put in place to ensure retention in care from the pediatric providers’ side, they are unable to ensure that their patients remain adherent and in care. Although they recognize patients who may be at an elevated risk for disengaging care after being placed in adult HIV care, they are unable to follow up, particularly in cases where patients are transferred to a different facility.

**Figure 2:**
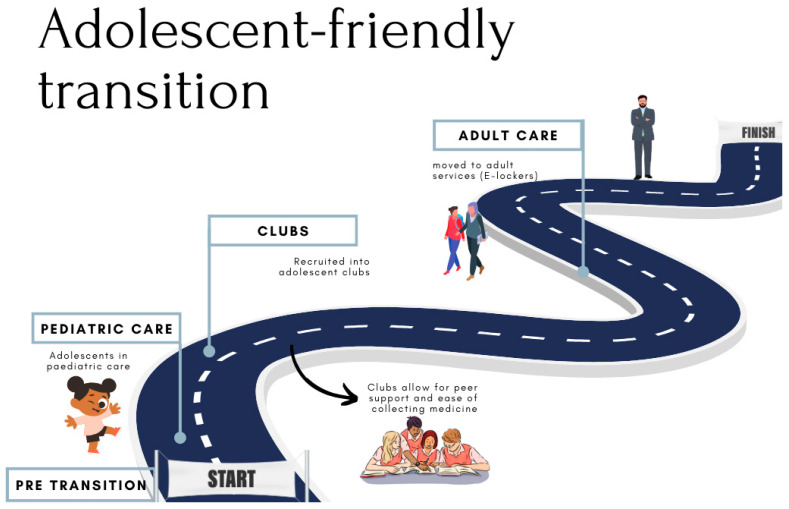
Adolescent-friendly pathway.

Figure 2 illustrates the *adolescent-friendly pathway* wherein facilities provide youth clubs or groups as a holding space for adolescents prior to transitioning them to adult HIV care. Here, adolescents are often seen by either a medical officer or a clinical nurse practitioner, who also facilitates the groups. The facility also has a dedicated space for adolescent groups. This category includes facilities that consider ALHIV’s readiness to transition as well as facilities that provide little to no information of the transition, where the groups are for medication collection purposes solely. In cases such as the latter, the providers maintain an active inquiry into the care being provided to adolescents.

As stated above, this transition process involves a middle ground between the transfer from pediatric to adult HIV services. This may involve a youth group or club, where adolescents have the opportunity to meet and speak with other ALHIV, that may be from the same area as them or provide a source of connection for them based on their experiences. Other than peer connection or support, this process also involves increased support from healthcare providers through providing a safe space at a time that does not hinder their schooling or personal lives. This is evidenced in the following:

*Yeah, HIV youth that I have. So, on their date for the appointment for the medication, they just come straight from their gate, straight nobody asks them anything. They just come straight to me. So usually on Mondays, I’ll prepare their medication. So, I will speak to them, and then send them to pharmacy. There is a specific person for them. Then she will prepare the meds for them, and then on Wednesday, I must go and fetch them the medication… Yes, if they want to come before they go to school, they can come. I’m going to give them medication. If they want to come after school, I’ll wait for them. But you wait until 10 past four, and they usually call me or WhatsApp me that I’m going to run late. So, can I come? Maybe I’m gonna arrive quarter past four then I can wait. Oh, they will tell me I’m not gonna come on today. I’ll come maybe on Friday, because it’s gonna take forever to come.*

(Participant L, clinical nurse practitioner)

Participant L explained how providing flexibility when the adolescents can come for their scheduled visit appeases them. This is supported by Participant A:

*And because we’re seeing 20 teenagers in one club, it’s more time-efficient from a clinical point of view.*

(Participant A, pediatric medical officer)

This shows that making the adolescents attend the clinic on the same day can be more beneficial. This is translated into their engagement in care as well, as Participant H mentions that “*they come back and they remain because there’s a difference between coming and not remaining*” (Participant H, clinical nurse practitioner). The connection between peers serves as another facilitator for not only remaining in the clubs, but in adherence as well, as evidenced in the following:

*So, one of the advantages of the club is that the teenager gets to meet another 15 to 20 young people who have HIV, and they figure out oh I’m not actually alone. This is actually a common thing. Um, it’s a shared experience.*

(Participant A)

Participant A discusses how perceived isolation may be mitigated through the adolescent clubs and assists in fostering a feeling of belonging. Moreover, adolescents are known to be avid social media users, and a part of the adolescent-friendly services being offered is connecting to peers and healthcare providers via social media messaging, as Participant L mentions: “*We have a WhatsApp group. This is only for the youth who is active now*”. This is strengthened by Participant H:

*They have access full access to me and* [the pediatric doctor] *because we have the clinic’s group chat whereby anyone can post really.*

(Participant H)

This participant explicates the availability and accessibility their patients are provided, which speaks to the World Health Organization’s definition of adolescent-friendly services [58]. However, to be able to participate in the adolescent clubs, there are clear criteria:

*There are clear criteria. Criteria really, we’ll look at your viral load. Viral load is the amount of virus in your in your blood. We want them to be virally suppressed. Yeah. There is a specific number that we look at. We want them to be virally suppressed. And they must be able to behave. They must be able to converse to make a conversation because they are going to be interacting with other kids.*

(Participant H)

Participant H explains the criteria necessary to be allowed into clubs; these criteria may inhibit access to this *beneficial* service that all adolescents may benefit from, despite their individual discrepancies. Furthermore, despite this middle ground being provided to adolescents, they are yet to transition to adult HIV services, and, while these groups may prepare them for adult care,

*I think really, by them, joining the youth club has really facilitated their growth. Them growing and accepting whatever circumstance and grow, personally and professionally.*

(Participant H)

This safe space may also make it difficult to move to a different, unfamiliar space. Participant L mentions, “*Very comfortable. I don’t think they will go*”. This strengthens the notion that, because adolescents have an increased accessibility of care currently, this may lead them to be hesitant to move to adult clinics, where the model of care may be vastly different. Participant L further supports this:

*They’re gonna say, I’m not ready to be out in the world… Did you see them? Do you think they can go there? I don’t think they will go there. Because I’m just telling them now, the other one is 23, just telling her now you will go to e-lockers soon. And she said, “I’m not going to”. So I doubt they will even go there.*

(Participant L)

Thus, the participants might not be confident in the adolescents’ retention in care after transitioning to adult care. Moreover, these facilities appear to lack a transition and post-transition plan, as Participant H explains:

*Ideally, they were supposed to be here until they are 24, but there is no active plan that is going to work perfectly well for them. We are still working on a plan whereby they really have to transition from 24, because really they are unable to attend each and every club, which then defeats the whole purpose of the club. So we are still looking into ways whereby they can really transition from this.*

(Participant H)

Thus, this transition is further delayed due to the healthcare providers’ hesitancy and uncertainty about adolescents being supported efficiently in adult HIV care.

**Figure 3:**
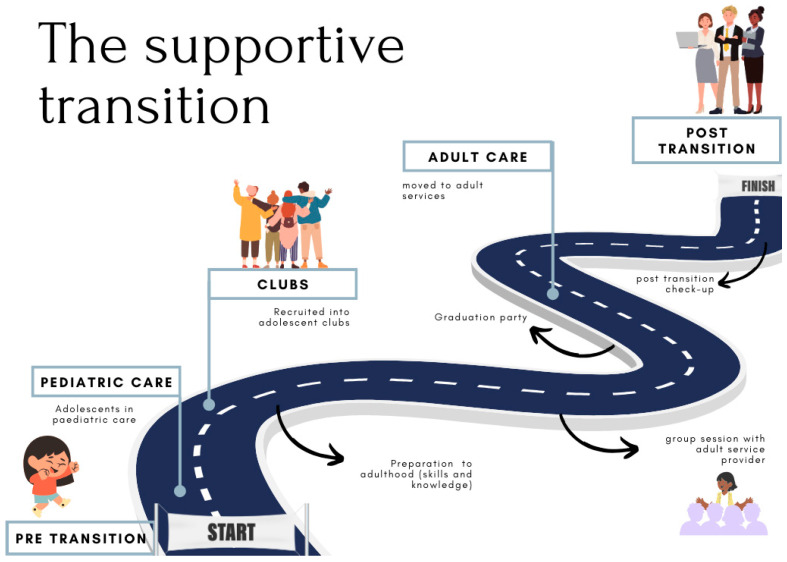
Supportive pathway.

The third transition process is the *supportive transition* pathway, as illustrated by Figure 3, where facilities provide pre- and post-transition support to adolescents. This includes youth clubs or groups, wherein skills and readiness are assessed and supported by healthcare workers and peer mentors. Moreover, post-transfer check-ups occur, wherein pediatric or adolescent service providers confirm the engagement in care for those they have transferred to adult HIV care.

This transition process involves a more holistic way in which care can be provided to adolescents across the continuum. Herewith, a strong relationship between the healthcare provider and the patient is also evidenced:

*No, he defaulted. And that time when he started defaulting, he had a CD4 count of 78, then he was away for a long time, and I thought, “Oh gosh”, and I kept on asking, where is he? And then when they do come back then, oh gosh, “I am glad to see you guys”.*

(Participant O, counsellor)

The healthcare provider therefore has the ability to get to know and remember their patients. Furthermore, this allows for a personal relationship to be fostered.

*You’re proud yes. Like that other one, right, he’s 18 now. He’s so beautiful, he’s a young man now, and see, they walk a long road with us. I always admire him, and I ask him, “is there a miss us?”*

(Participant O)

Participant O mentions how this developing relationship allows the provider and patient to speak on a comfortable level. As clinical nurse practitioners or counsellors, they can see the same patients before and after they have transitioned—granted this transition happens within the same facility—as Participant N mentions, “*No, we see them grow up*”. (Participant N, clinical nurse practitioner). Similarly, Participant I mentions, “*They grow with us*” (Participant I, counsellor), strengthening this lifelong support a patient can receive from the facility. Moreover, this allows the provider to identify potential issues with their patients:

*As a result, we do have a social worker who comes and does the sessions with us. She was a counsellor. And then she upgraded to the social worker. And then also some of the kids that have social issues. It’s very easy for them to see that. Oh, this is [the social worker] I know from my area now she is here, so she helps a lot. So, for us in the clinic, they’ve got a huge support. For them, it’s not actually just a way of growing with us. So, the support is solid.*

(Participant I)

Adolescents have an increased sense of support at these facilities. As mentioned, these facilities are also able to provide adolescents with support groups comprising peers. A perceived benefit for the adolescents is the planned topics that are geared towards preparing them for their lives outside of their chronic illness:

*Uh, we, we kind of sit in a circle but order some chips or something from Checkers online. And then we just chat, meet and greet, say hi. Um, and then, you know, book another visit. And what I also do with the first session is I kind of gauge what they want. So, I do a little box, and I put their suggestions in the box. Um, what they would like to talk, what they want from the group. Okay. So so then I take that and I read it, and I kind of make up a plan for the year. Yeah. Well, I plan the sessions accordingly.*

(Participant B, medical officer)

Participant B discusses how they use the input from adolescents to make the care they provide during the groups more adolescent-focused. This is strengthened by Participant G, discussing how these sessions may benefit the adolescents:

*So, so this so they, they come to, so they come together as a group, they, we’ll, we’ll try and have sessions with them sometimes that’s just, playing games together. Sometimes it’s initiating discussions. Like yesterday they had a discussion around a sort of that ended up sparking into a discussion about how they support each other taking their treatment. Sometimes it will be a planned activity. So, we’ve got sort of various planned activities, particularly with the younger ones when they first come into club around sort of feeling comfortable about talking about HIV, understanding, about, taking their treatment and, how it works.*

(Participant G, pediatric medical officer)

Therefore, the clubs may allow the adolescents to not only foster relationships, but to increase their knowledge on their treatment-taking and chronic illness. The healthcare workers are also able to more readily identify possible patients who may disengage in care:

*So, so you know who’s missing when the club comes. We know who’s missing. We try and contact them. We develop relationships with them.*

(Participant G)

Linkage to care may be increased as a result of this strong relationship and access to their information. This also involves certain criteria to be met when enrolling adolescents into these clubs:

*There’s like criteria to being enrolled in the club. And so things like this closure, family support, mental health stability, um, viral load suppression, things that you kind of want before they go into the club.*

(Participant B)

Participant G strengthens this:

*So, the criteria for the club are that you’re over ten, that you’re fully disclosed, and that you are virally suppressed at the time of. Entry into the club. And however, when you’re in the club, we try and keep you even if you become unsuppressed, even if you get pregnant. Even if you get TB. Even if you default, so we try to keep them because we found early on that if we worked with the adult club model, we lost teenagers who didn’t know what’s happening with them.*

(Participant G)

Despite the set criteria, this club attempts to keep the adolescents linked, regardless of any obstacle they experience in their treatment-taking. Moreover, the groups may assist in preparing adolescents to transition into different life stages:

*So they come here on Fridays and if they are in the age groups booked, so 5 to 9, they kind of will get used to each other and play with each other and the transition, because at the moment we are having issues with we have the club adherence club, but they don’t always want to come to that group for reasons that they might see someone that’s at school with them. So, the disclosure then happens when you’re in the group. Yes. Like if you walk in. Yeah. Yeah. So, if they and if they are booked in their age groups, they’ll grow up with each other and then it will just be easier to transition into the club.*

(Participant B)

Previous research indicates that, oftentimes, adolescents often hesitate to remain in adult care due to concerns about being recognized by someone familiar, and unintentionally disclosing their status [7,12]. Becoming familiar with a group of peers may diminish the fear of disclosure and perceived stigma. Furthermore, through this, Participant B mentions that it can be helpful in easing into adult care: “*So it is important to transition and identify and cushion the blow of adult care*”. Participant I reinforces this sentiment:

*So, we speak with them openly about taking care of their lives, taking the treatment. So, I think from eight years, seven, eight up until they are teenagers, they are adults. And then we, we transfer them to the adult club.*

(Participant I)

Thus, this approach supports the preparation for the autonomous management of treatment. However, as adolescents are found to be less likely to remain in care consistently, this is also a barrier for the adolescent clubs:

*You’ve got a 25 to 50% loss to follow-up rate in the area for ARVs in the 15–24 age bracket. We’ve got 30%. Defaulting on TB. Genuine. So, we that’s what we’re up against. But that that’s why we keep hoping That these small. This small group of teens, we try and help them to just navigate and to stay through. As much as much as we can.*

(Participant G)

The challenge of keeping adolescents in care remains, despite the positive uptake in the groups. However, Participant G maintains that the clubs are important for adolescents:

*We now have a dedicated club room, which is different to where we were before. But now we’re able to see them as a group again. And we’re trying to build that cohesion as a group because the peer that, that peer is getting to know that there are others on this journey with me, is a huge part of, I think it’s a huge part of, of. The clubs as the clubs grow. And so those relationships, I think, can start to become quite important.*

(Participant G)

This is supported by Participant I, stating that “*all of our kids to go to teenage life and they will come back*”. This shows that, despite the challenges the adolescent period may face, the service they provide at these facilities is beneficial for adolescents to remain in care.

This *supportive transition* process is strengthened by ensuring adolescents are ready to transition, as Participant G mentions that the transition is not a “*once-off event*”:

*But, but, you know, but certainly a service that is quite supportive and perhaps a bit more flexible to transition and go on that journey into a service where actually it’s much more hands-off and you have to take much more responsibility. It’s a journey from one place to the other and how we equip them to kind of get there and manage and when they don’t like it.*

(Participant G)

Therefore, as this is a *process*, it requires a comprehensive approach whereby adolescents are adequately prepared to manage their care on their own. Moreover, Participant G posits how making the transition as a group process may also be beneficial, particularly in ensuring preparedness and readiness within the group as a whole:

*I think generally the idea of transitioning them as a group is that most will be ready. So, we would transition them as a group. So, the last time we had the whole six months, we had like a, a period of like telling them. So, we’re now telling them later this year. So, the group yesterday and you know, we’re going to be transitioning you to the adult service later this year and the second half of this year. This is the plan because you’re all big now you know, so we’re starting that process. And then what I did last time was then we did actually get the nurse to come see the in the club. And then the other thing we did is we had like a graduation a graduation braai which not so many people came to but it was a very nice event. And we actually had like, a braai to say, you know, this is it where, you know, bye! We are still here. You’ll still see us. We will still wave at you in the clinic.*

(Participant G)

Adolescents were not only prepared, but they were also clearly given a clear understanding of how the process will work with the reassurance that they will not be “abandoned” by their current healthcare providers. She further posits that having a post-transition follow-up is also helpful, particularly in identifying any possible disengaged adolescents:

*And by engaging the staff member who was going to be receiving them and then following up that with the register. So what I did is just check through the register, thought who came, who didn’t come. So we still provided some follow-up with calling if they didn’t come out so that they came within the time.*

(Participant G)

Therefore, this *supportive transition pathway* allowed for pre-, during-, and post-transition support being provided to adolescents to ensure that they are prepared and ready, and have the necessary skills to manage their care. It is important to note that these adolescents are in dedicated clubs, and do not reflect the cohort that is not enrolled in the clubs.

## 4. Discussion

The successful transitioning from pediatric to adult HIV care for ALHIV requires an optimal process that acknowledges the unique and dynamic needs of adolescents across time and space [59]. Factors such as transition readiness, psychosocial support, appropriate levels of self-disclosure, and the attainment of self-management skills of ALHIV are paramount [18,39,60,61]. The role of healthcare providers in the transition process is critical as evidence shows that adolescents need appropriate health education in pediatric services (pre-transition) [10,12,62,63], psychosocial support during the transition [33,34,62,63], and follow-up in the adult care program post-transition [10,34,63].

In the pre-transition period, it is essential that we assess the transition readiness of ALHIV on ART, to ensure they are sufficiently prepared to face the challenges of autonomous treatment and responsible self-management [10,35,62]. It is evidenced that inadequate preparation for transition can lead to hesitancy. This hesitation stems from a fear of losing meaningful relationships with peers and current healthcare providers, overcrowded adult clinics, and the inability to come to the clinic as adult schedules are less flexible than what they received in pediatric care [10,12,62].

In the *transfer-only* pathway, we note that no readiness assessment is carried out. Instead, the decision to transition an ALHIV to adult care is based primarily upon the consideration of age—where the common practice is to transfer pediatric HIV patients when they reach 12–13 years old [39]. This may, in part, be explained by the lack of facility infrastructure and pediatric healthcare providers as well as an expectation that adult HIV programs can accommodate higher patient loads [36,39,61,64]. Thus, in this pathway, ALHIV are moved away from a more individualized, caring environment to a service where they need to be responsible for their own care, with no consideration of their adolescent-specific needs as they are treated as adults [59]. Furthermore, current, available guidelines in this context are more medically focused by only providing guidance on transitioning ALHIV to the adult treatment regime, with an absence of guidance on services and care specifically geared toward the entirety of the transition to adult HIV services for ALHIV [27,28]. The implications of this include adolescents being transitioned to adult care without achieving optimal readiness. In the *adolescent-friendly* pathway, adolescents are prepared for the transition through youth clubs or other psychosocial support measures. This is also evidenced in the *supportive transition* pathway, wherein the transition is discussed in the group sessions and adequate preparation for the physical transfer is carried out. The last-mentioned pathway improves the chances for a successful transition and positive post-transfer outcomes for the adolescent, since it is predicated on ALHIV having a sense of readiness to transition and [treatment] self-efficacy [12,37].

The transition process requires that ALHIV receive more support, to counter the trend of decreasing adherence and retention in care in the adolescence period. This support should ideally be provided by both healthcare providers and peers, and within a space where their hesitancy to transition and need for growth in their knowledge of HIV and HIV care are acknowledged [33,35,63]. More specifically, the support for transition should include information about the process from their healthcare providers, psychosocial support, and an introduction to adult services/providers. This is evident in the *adolescent-friendly* and the *supportive transition* pathways, through the provision of an adolescent space with youth groups and dedicated healthcare providers. It is reported elsewhere that services that are adolescent-friendly does not interfere with school attendance through the scheduling of appointments after school, and minimizing lengthy clinic visits [7,34]. The *transfer-only* pathway lacks almost all forms of transition support mentioned above. As seen in the transition mapping survey, these facilities house a large number of ALHIV but lack dedicated services for adolescents. As such, adolescents are “aged out” of adolescent care when they are legally adults at the age of 18 years, enter employment or tertiary education, or, in the case of females, fall pregnant. It is widely reported that, in facilities that apply the transfer-only pathway, pediatricians tend to hold on to ALHIV in pediatric care until forced to transfer them to adult care.

The *adolescent-friendly* pathway addresses many of these gaps left by increasing the level of peer engagement and providing psychosocial support for adolescents. Peer support, in addition to routine care, has a positive impact on ALHIV’s adherence and retention in care, as well as improving self-autonomy in managing their care, which, in turn, bodes well for post-transition health outcomes [18,65]. Moreover, it is reported that feelings of isolation when transitioning, negative feelings about being transferred to adult services, negative feelings about living with HIV, and perceptions of stigma all play negative roles in the adolescents’ experience of living with HIV [15,37,38,41]. Through peer support, the feelings of isolation and negative perceptions of transitioning may be mitigated, particularly in adult care settings. Furthermore, the clubs are a convenient way to collect medication for adolescents, where they do not have to wait in a queue as they would at adult clinics. Another benefit of providing care to adolescents through adolescent groups or clubs is that these groups occur at times befitting their schedules, i.e., after school. It is particularly important that the compromise between school attendance and healthcare is considered as it is found to be a barrier to retention in care for ALHIV [7,15]. Thus, the groups that are held outside of school hours mitigate this risk of losing retention in care and adherence to pick-up schedules.

The *supportive transition* pathway follows a holistic approach to the transition, which acknowledges that the transition as a process requires support prior to, during, and after its occurrence. Transition practices which prioritize communication from and between healthcare workers, psychological and social support, group transition, mHealth, education, and individualizing care plans may engender more positive health outcomes after transitioning to adult care, as it induces a more welcoming and inclusive environment [22]. Therefore, this pathway holds benefits in providing care for ALHIV, particularly during the transition. The structured peer group sessions, which are often facilitated by a trained healthcare worker, facilitate ALHIV’s self-management skills and knowledge of their illness. In the supportive transition model, ALHIV remain in their peer groups post-transfer to adult services, which further improved the monitoring of treatment outcomes post-transfer and the follow-up of those who disengage from care. The example of the Zvandiri Trial [66] illustrates how peer support can be implemented through peer-led programs, with adequate training and ongoing support for peer supporters. Thus, peer groups are sustained throughout the transition process and into post-transfer as young adults [19,66,67]. Zanoni et al. [30] developed an mHealth tool that incorporates both peer support and an opportunity to increase HIV knowledge and promote adherence and retention in care in the South African context, which could lead to improved transition outcomes. Additionally, adolescents living with chronic conditions need to be equipped with the necessary knowledge about their condition, and general healthcare management [4]. As adolescents have greater access to information, and their healthcare providers, this may have a positive impact on their motivation to remain adherent, and, ultimately, transition successfully [12,15,68]. It is important to note that the facilities that do provide the *supportive transition* pathway are primary-level healthcare facilities that are financially supported by NGOs, or have additional resources, and, therefore, may not be replicable in the resource-constrained settings in South Africa.

Post-transition has been found to be a period of low adherence and engagement in care for ALHIV, if not carefully and supportively monitored [19]. Educational support can and should be provided to ensure that the adolescent is informed about adult clinic operations and who their healthcare providers are [12,19,24]. The findings of this study show that the *supportive transition* pathway solely provides dedicated attention to the transition as a process as well as post-transition follow-up. As mentioned previously, the facilities that implement the *transfer-only* pathway transition ALHIV with no preparation and guidance. They can be characterized as facilities with a high patient load and lack of resources and infrastructure, and, therefore, not having the logistical capabilities to follow adolescents post-transfer, regardless of the healthcare provider’s interest in doing so. Despite the benefits of the *adolescent-friendly* pathway of transitioning ALHIV, the lack of a structured transition protocol and post-transfer support run the risk of reversing the benefits that they received as adolescents in the youth clubs. The results from our transition mapping survey indicated a lack of a formalized, written transition protocol across most participating facilities, and a lack of transition-specific policy guidelines in South Africa. This is supported by the existing literature, indicating that there is a sparsity in transition policies and protocols at both the national and clinical levels [22]. Within the South African context, it is necessary to acknowledge the deficiency of transitioning ALHIV to adult HIV care, and the lack of guidelines on transitioning, as well as the lack of awareness of this policy amongst healthcare providers, as evidenced in this study.

One of the limitations of this study is the small sample size, which may impact the extent to which the transferability of the findings to other settings in South Africa may be considered. The sample consisted of healthcare workers and does not reflect the lived experiences of ALHIV within these pathways of care undergoing the transition to adult HIV care. However, this study sample included participants and healthcare facilities across different levels of care and variable resource contexts and, therefore, provides insights into the dominant practices in which transitioning ALHIV to adult care in the Cape Town Metro takes place. The selected facilities and health workers were all situated in high-density urban areas—thus, the comparability of transition practices may be very low with regard to rural settings in the rest of the Western Cape province and South Africa. However, the findings of this study may resonate with other high-density metropolitan districts in South Africa, where facilities are characterized by high HIV-patient loads, particularly among adolescents.

## 5. Conclusions

Mapping transition pathways can be beneficial in highlighting how facilities, based on their available resources, can offer a more optimized transition process. Thus, optimizing the practices of transitioning ALHIV would ultimately lead to successful health outcomes for ALHIV in terms of optimal adherence and persistent engagement in care [10,40,41]. The findings from this study delineate three distinct pathways in which the transition from pediatric to adult HIV care may take place. The distinctions are made through the type of service being provided to ALHIV and available resources and staff. There is a paucity of research that maps the pathways of how the transition for ALHIV takes place [25,39]. The optimal or successful transition to adult HIV care includes ALHIV being able to effectively and independently seek medical care, manage appointments, and access their medication and insurance, and the specific considerations regarding their chronic care management [59]. Two of the pathways (*adolescent-friendly* and *supportive transition)* provide pre-transition support to ensure transition readiness. Care and support are further required as adolescents are being transitioned, and both of the abovementioned pathways provide this through youth groups (clubs) and psychosocial support. Only the *supportive transition* pathway provides a post-transition procedure wherein support is provided to transitioned ALHIV to ensure a linkage to care and retention in care.

Whereas the *supportive transition* pathway facilitates the most optimal process for the attainment of a successful transition of ALHIV on ART, it is acknowledged that this kind of offering requires considerably more resources, which is not present in most public healthcare facilities, since it is provided by external agencies. Therefore, we recommend a greater public investment in rolling out adolescent-friendly pathways in the delivery of care to ALHIV and expanding these services to include the monitoring of ALHIV post-transition. Subsequently, models of care that incorporate peer-led groups and clubs may offer a more cost-effective solution, as well as the utilization of mHealth to complement the *supportive transition*. Such an investment should ideally be accompanied with the development of relevant health policies and implementation guidelines that would signal political will and provide the impetus and agency of implementation at the service level and in other settings in South Africa. We, therefore, recommend additional human resource support and the integration of electronic monitoring systems, and the use of mHealth to maintain the adherence and engagement in care of ALHIV post-transition and as young adults. There is an urgent need for the development of transition protocols aimed at improving service delivery for ALHIV who are transitioning to adult HIV care. Particularly, in prioritizing adolescents receive adequate pre-transition support such as preparation and education, and support during the transition, which includes peer support initiatives and increased communication between pediatric and adult healthcare providers, and post-transition, such as a follow-up to ensure retention in care and continued peer support.

## Figures and Tables

**Table 1:** Site characteristics (N = 6).

Facility	Level of Care	Number of ALHIV on ART
1	Primary	156
2	Primary	195
3	Primary	323
4	Secondary	80
5	Primary	100
6	Tertiary	90

**Table 2:** Characteristics of adolescent-specific services (N = 6).

Category	n	Type of Facility/Level of Care
ALHIV clinic	4	Primary and secondary
ALHIV support group	5	Primary and secondary
**Separate adult and pediatric facility (n = 3)**		
Routine practice for ALHIV transition	3	Primary, secondary, and tertiary
Written transfer protocol	0	
Pediatric–adult clinic communication during transition	3	Primary, secondary, and tertiary
**Method of adult–pediatric communication (n = 3)**		
Verbal only	2	Primary and secondary
Written only	0	
Both	1	Tertiary
**Post-transfer follow-up by pediatric facility n = 2)**		
Contact parent/ALHIV	1	Primary
Contact adult clinic	0	
Contact parent/ALHIV and adult clinic	0	
None	1	Secondary
**Transfer criteria (n = 3)**		
Age	2	Secondary and tertiary
Disclosure	0	
Readiness assessment	2	Primary and tertiary
HIV knowledge	1	Tertiary
Pregnancy	1	Tertiary
Marriage	0	
CD4 count	0	
Viral load	2	Primary and tertiary
Other: Transition of teen club as a whole into an adult club	1	Primary

## Data Availability

The raw data supporting the conclusions of this article will be made available by the authors upon request.

## Supplementary Materials

The following supporting information can be downloaded at: https://www.mdpi.com/article/10.3390/tropicalmed10010005/s1, File S1: Interview guide for healthcare workers.
