# Supplementary material for: Mapping the Transition of Adolescents to Adult HIV Care: A Mixed-Methods Perspective from the Cape Town Metropole, South Africa
Source: Trop Med Infect Dis. 2024 Dec 24;10(1):5. doi: 10.3390/tropicalmed10010005 (PMC11768539; doi:10.3390/tropicalmed10010005)
Supplement: Supplementary file 1 [file tropicalmed-10-00005-s001.zip › tropicalmed-3349299-supplementary.pdf]

Appendix 2: Interview guide for phase 2

(This interview guide is translated into Afrikaans and will be translated into isiXhosa)

Aim: To describe healthcare practices and policies on transitioning of ALHIV to adult HIV care in the Cape Town Metropole and South Africa, respectively (Phase 2).

Demographic data

Name:

Age:

Gender:

Language:

Area of work:

Position of occupation:

Years working with ALHIV:

Guiding questions:

1. What is the current available adolescent-specific services for ALHIV at your facility?
2. What is your understanding of the transition for ALHIV from paediatric to adult care?
3. How does this transfer from paediatric to adult care for ALHIV take place where you work?
4. How is adult HIV care different from paediatric care?
5. Do ALHIV adhere better to their HIV care in paediatric care or adult care?
6. In your experience, what do ALHIV struggle most with after they have transitioned to adult care?
7. What would you say are the most salient barriers to adherence and retention in care for ALHIV who have transitioned to adult care?
8. What are the current needs of your facility that could benefit ALHIV who are yet to transition to adult care?
9. What are the current needs of your facility that could benefit ALHIV who have already transitioned to adult care?

Healthcare manager-specific questions:

- What are the current guiding policies in the WCDOHW for transferring ALHIV to adult care?

- Are these guiding policies different from national policies?
- In which ways are transitioning to adult HIV care different provincially compared to nationally?
